# Improved Production and In Situ Recovery of Sesquiterpene (+)-Zizaene from Metabolically-Engineered *E. coli*

**DOI:** 10.3390/molecules24183356

**Published:** 2019-09-15

**Authors:** Francisco Aguilar, Thomas Scheper, Sascha Beutel

**Affiliations:** Institute of Technical Chemistry, Leibniz University of Hannover, Callinstr. 5, 30167 Hannover, Germany; aguilar@iftc.uni-hannover.de (F.A.); scheper@iftc.uni-hannover.de (T.S.)

**Keywords:** (+)-zizaene, khusimene, khusimol, vetiver essential oil, in situ product recovery, expanded bed adsorption, sesquiterpenes, terpenes, *Chrysopogon zizanioides*

## Abstract

The sesquiterpene (+)-zizaene is the direct precursor of khusimol, the main fragrant compound of the vetiver essential oil from *Chrysopogon zizanioides* and used in nearly 20% of men’s fine perfumery. The biotechnological production of such fragrant sesquiterpenes is a promising alternative towards sustainability; nevertheless, product recovery from fermentation is one of the main constraints. In an effort to improve the (+)-zizaene recovery from a metabolically-engineered *Escherichia coli*, we developed an integrated bioprocess by coupling fermentation and (+)-zizaene recovery using adsorber extractants. Initially, (+)-zizaene volatilization was confirmed from cultivations with no extractants but application of liquid–liquid phase partitioning cultivation (LLPPC) improved (+)-zizaene recovery nearly 4-fold. Furthermore, solid–liquid phase partitioning cultivation (SLPPC) was evaluated by screening polymeric adsorbers, where Diaion HP20 reached the highest recovery. Bioprocess was scaled up to 2 L bioreactors and in situ recovery configurations integrated to fermentation were evaluated. External recovery configuration was performed with an expanded bed adsorption column and improved (+)-zizaene titers 2.5-fold higher than LLPPC. Moreover, internal recovery configuration (IRC) further enhanced the (+)-zizaene titers 2.2-fold, whereas adsorption velocity was determined as critical parameter for recovery efficiency. Consequently, IRC improved the (+)-zizaene titer 8.4-fold and productivity 3-fold from our last report, achieving a (+)-zizaene titer of 211.13 mg L^−1^ and productivity of 3.2 mg L^−1^ h^−1^. This study provides further knowledge for integration of terpene bioprocesses by in situ product recovery, which could be applied for many terpene studies towards the industrialization of fragrant molecules.

## 1. Introduction

The biotechnological production of chemicals by engineered microorganisms is a potential alternative for the production of terpenes from renewable resources [1]. Recent advances in metabolic engineering have made possible the production of terpenes by microbial platforms at economically-feasible titers (over grams of terpene per liter of broth), reaching the industrial scale, such as artemisinin, β-farnesene, and squalane [2,3,4]. Fragrant sesquiterpenes used in the cosmetic industry are potential candidates to be produced by biotechnological systems, such as the sesquiterpenes contained in the vetiver essential oil (VEO) from the grass *Ch. zizanioides*. VEO is an important component for the formulation of cosmetics and it has been used in nearly 36% of Western perfumes and 20% of men’s fragrances [5] with a total world production of 300−350 tons per year [6]. VEO is composed of a mixture of sesquiterpenes and their hydroxylated derivatives, with a characteristic dark woody scent granted principally by khusimol, its main fragrant component [7,8]. Moreover, the biotechnological production of khusimol could lead to a reliable supply for the cosmetic industry and to avoid the shortages from the traditional supply of VEO (extracted from the vetiver roots) due to natural disasters such as earthquakes and floods [6,7].

An important step towards the microbial production of khusimol was the whole-cell bioproduction of (+)-zizaene (syn. khusimene), the direct precursor of khusimol, by a metabolically-engineered strain of *E. coli* [9]. This was demonstrated by engineering the mevalonate (MEV) pathway to increase the levels of the natural sesquiterpene precursor E,E-farnesyl diphosphate (FDP) and overexpressing the (+)-zizaene synthase (ZS) [9]. The latter terpene synthase catalyzes the substrate FDP through a complex reaction by carbocation rearrangements and cyclizations to yield the tricyclic (+)-zizaene with a product specificity over 90% [10].

Further development of the (+)-zizaene bioprocess would comprise the scale-up of the fermentation to bioreactors (upstream process) and the product recovery (downstream process). As usually, the scale-up of the fermentation is performed by optimizing the bioreactor variables such as pH, oxygen supply, and stirring [11]. Additionally, scale-up studies can be performed using fed-batch fermentation by feeding of a carbon source, such as glucose, to reach high cell density cultures (HCDC), as is the case with the production of farnesene, santalene, cucurbitadienol, and (-)-α-bisabolol [12,13,14].

The downstream process is one of the main challenges towards the industrialization of terpenes because of their inherent physicochemical properties such as hydrophobicity and volatility [15]. Such bioprocesses often suffer cell toxicity, product inhibition, product volatilization, and product degradation, which can be devastating for the bioprocess productivity [16]. As an alternative, in situ product recovery (ISPR) has been applied for the downstreaming of terpenes, defined as the removal of product during its formation in the reactor [17].

The liquid–liquid phase partitioning cultivation (LLPPC) is the most used ISPR technique for terpene recovery, in which the fermentation is carried out with a liquid extractant, forming two immiscible phases [15]. The liquid extractants are usually bio-compatible organic solvents with a high log P value that do not partition into the microbial membrane and sequester the terpenes from the aqueous phase [18,19]. Because of the ease of LLPPC, it is the first choice for ISPR of terpenes and it has been applied in the recovery of many terpenes such as limonene [20], taxadiene [21], α-santalene [22], and amorpha-4,11-diene [23].

Another ISPR alternative is the solid–liquid phase partitioning cultivation (SLPPC), in which a solid extractant is in contact with the fermentation, such as polymeric adsorbers, porous resins, zeolites, or activated charcoal [24]. Due to their high affinity to hydrophobic compounds, these extractants can selectively adsorb terpenes and can be applied in numerous configurations because of their mechanical stability [15], as implemented for the production of perillylic alcohol [25], epi-cedrol [26], and linalool oxides [27]. 

**Figure 1:**
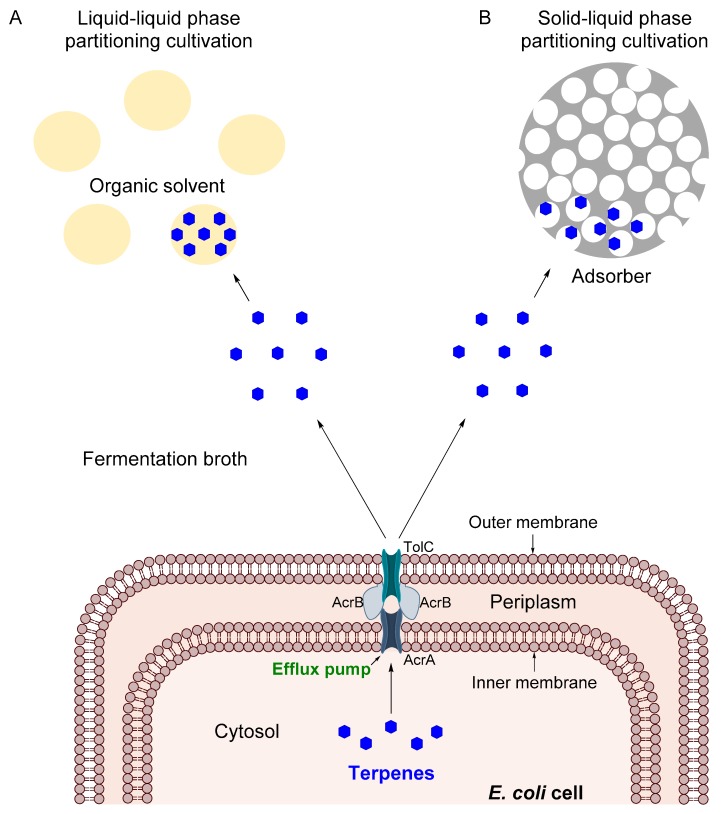
The mechanism for the in situ recovery of terpenes produced from metabolically engineered *E. coli*. (**A**) Liquid-liquid phase partitioning cultivation is carried out by liquid extractants (organic solvents), which extract the terpenes by hydrophobic interactions. (**B**) Solid-liquid phase partitioning cultivation utilizes solid extractants (adsorbers) and recovers the terpenes by adsorption. The tripartite efflux pump *AcrAB-TolC* is used as an example of a secretion system for hydrophobic molecules and its components are properly described.

The mechanisms for ISPR of terpenes in *E. coli* are described in Figure 1 for both LLPPC and SLPPC. Accordingly, terpenes are synthesized in the cell cytosol and exported to the fermentation broth by solvent-resistant tripartite efflux pumps such as *AcrAB-TolC* and *MdtEF-TolC* [28,29]. For the LLPPC, terpenes are extracted into the organic solvent by the principle of hydrophobic interactions, in which non-polar hydrocarbons form aggregates together and separate from the aqueous phase [30]. In the case of SLPPC, terpenes are adsorbed to the porous surface of the solid extractant whether by physisorption or chemisorption, forming a film in the porous surface of the extractant, without altering the terpene structure [31]. Eventually, factors such as the chemical structure of the adsorber, pore size, particle size, and specific surface area will affect the adsorption efficiency [32]. 

Regardless of whether LLPPC or SLPPC is used, the configurations for ISPR of terpenes can be applied internally or externally in a direct or indirect approach [33]. Internal recovery configuration (IRC) is based on the extractant phase inside the reactor vessel, whereas in the external recovery configuration (ERC), the fermentation broth is recirculated towards an external loop to an extractant unit, such as an expanded bed adsorption (EBA) column [16]. In consequence, the choices for ISPR of terpenes are vast, whereas commercial processes employ a single or a combination of ISPR techniques, including adsorption recovery and/or gas phase recovery (gas stripping) [34]. However, the choice of configuration depends greatly on the downstream cost, which is determined by several factors such as the cost of extractant, number of phases, and recovery process, which will be related to the properties of the target molecule [33,35]. Moreover, ISPR provides numerous advantages when compared to the traditional extraction of terpenes and contribute towards a greener chemistry by reducing the recovery stages, amounts of solvents, and extracting products under milder conditions, which results in the reduction of the downstream costs [36].

In our previous report, we demonstrated the production of (+)-zizaene by a metabolically-engineered *E. coli* and optimized the fermentation conditions at shake flask scale, comprising the induction, media, pH, and growth temperature, reaching a (+)-zizaene titer of 25.09 mg L^−1^ and a productivity of 1.05 mg L^−1^ [10]. However, the scale-up for the bioprocess of (+)-zizaene requires a suitable ISPR configuration to circumvent product loss and further improve the recovery of (+)-zizaene.

In this study, we developed an integrated ISPR configuration for the microbial production of (+)-zizaene at a 2 L bioreactor scale with adsorber extractants in order to improve the (+)-zizaene recovery. For that, the product loss by volatilization was analyzed and the (+)-zizaene recovery was improved by using LLPPC at shake flask scale. Furthermore, distinct polymeric adsorbers were evaluated, in terms of selectivity and recovery ratio, as potential extractants to be applied at bioreactor scale. The desorption process was studied by a comparative assessment of organic solvents. The bioprocess was scaled up to 2 L bioreactors using the fed-batch culture technique and three ISPR configurations by direct contact mode were tested: ERC, IRC, and IRC with gas stripping. As a result, the (+)-zizaene titers and productivity were improved significantly.

## 2. Results

### 2.1. Product Volatilization Measurements and (+)-Zizaene Recovery by LLPPC

In our previous report, we demonstrated the microbial production of (+)-zizaene by engineering the MEV pathway and the ZS synthase in a multi-plasmid *E. coli* strain [9]. To further improve the production of (+)-zizaene, the bioprocess development requires the scale-up to stirred-tank bioreactors to reach HCDC, and an efficient downstream procedure. However, the recovery of terpenes involves special considerations due to their physicochemical properties. For the production of (+)-zizaene, loss of product could be expected during cultivation due to volatilization or microbial degradation. Moreover, toxicity to the *E. coli* cells could occur because of (+)-zizaene accumulation in the fermentation. 

**Figure 2:**
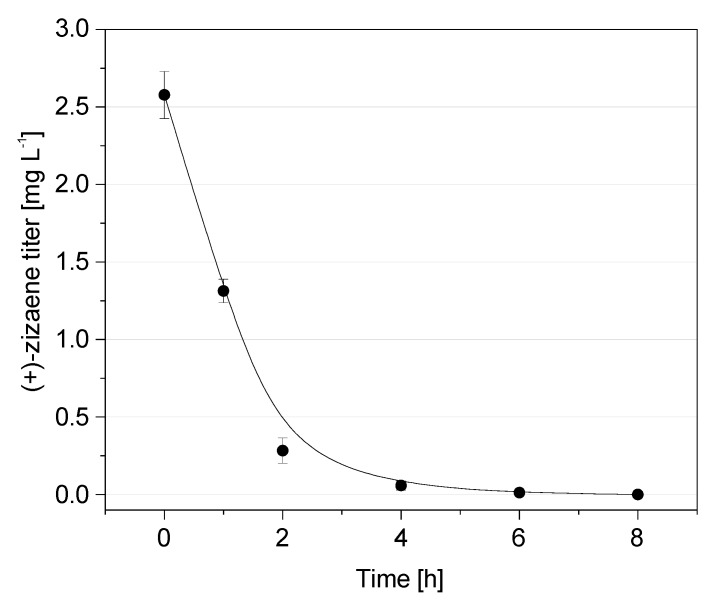
Loss of (+)-zizaene by volatilization on cell-free media at shake flask scale. Data are the mean of three replicates with error bars representing the standard deviation (SD).

Initially, we analyzed the loss of (+)-zizaene at shake flask scale without extractants by cultivating an induced *E. coli* TZS+MevZS strain, grown for 24 h. After removing the cells from the culture broth, (+)-zizaene measurements were taken time-wise from cell-free media. As a result, half of the (+)-zizaene amount was volatilized after 1 h and only traces were detected after 4 h (Figure 2). This demonstrates that, as for most terpenes, volatilization is a major constraint for the (+)-zizaene production. Eventually, the application of ISPR could provide solutions for these shortcomings, taking advantage of the hydrophobicity of (+)-zizaene (log P = 5.10), which could be extracted simultaneously during cultivation whether by liquid- or solid-phase recovery. 

**Figure 3:**
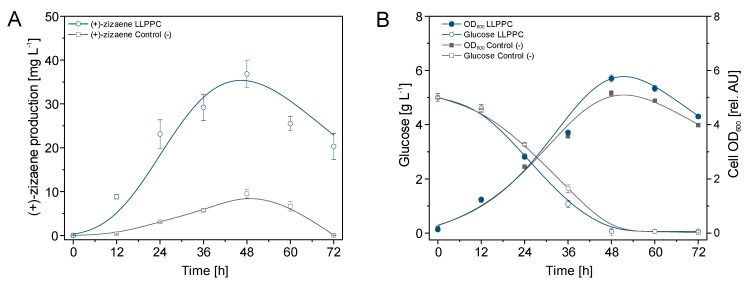
Comparison of the (+)-zizaene production (**A**) and growth kinetics (**B**) between the liquid-liquid phase partitioning cultivation (LLPPC) and the control (-) without extractant from the *E. coli* TZS+MevZS strain. Plots correspond to the mean of three independent experiments with error bars as SD.

To prove these hypotheses, a comparative test comprising a LLPPC with a solvent overlay and a negative control (without extractant) was performed with the *E. coli* TZS+MevZS strain at shake flask scale. As shown in Figure 3A, the (+)-zizaene production on the LLPPC and negative control tests followed similar kinetics, reaching the maximum peak at 48 h and dropping afterward. The LLPPC was nearly 4-fold higher when compared to that of the negative control at the highest production peak. Therefore, the loss of (+)-zizaene was estimated at nearly 27 mg L^−1^ due to volatilization at 48 h. Similarly, cell growth was higher on the LLPPC (OD_600_ 5.7 and biomass 2.1 g_DCW_ L^−1^) than the negative control (OD_600_ 5.1 and biomass 1.7 g_DCW_ L^−1^) at 48 h, suggesting a toxic effect due to (+)-zizaene accumulation in the control tests (Figure 3B). 

### 2.2. Screening of Polymeric Adsorbers for SLPPC

Although LLPPC has been used widely for isoprenoid recovery, SLPPC with solid extractants can be advantageous towards the scale-up of bioprocesses due to the following features: reusability, bio-compatibility, cost reduction of organic solvents, non-emulsion formation, and simple separation from the aqueous phase [15,37,38]. Thus, an in vivo adsorber screening analysis was carried out based on Halka [11] at shake flask scale. The tested polymeric adsorbers were chosen according to their affinity to adsorb hydrophobic molecules, as demonstrated in previous terpene recovery studies [25,39]. A negative control without extractants and a LLPPC control were also included. 

As a result, the adsorbers showed similar product selectivity, where the terpene profile for all the tested adsorbers by GC-MS showed approximately a product ratio of 90% of (+)-zizaene, 9.5% of β-acoradiene, and traces of hydrocarbons (Figures S1 and S2). 

**Figure 4:**
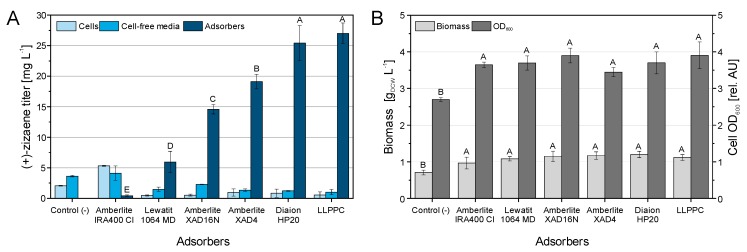
Screening of distinct polymeric adsorbers for the recovery of (+)-zizaene. (**A**) (+)-zizaene titers recovered from cells, cell-free media, and adsorbers. (**B**) Cell growth expressed by biomass and cell optical density. The negative control corresponds to culture without extractants. Data are the mean of three replicates with error bars as SD. Variables analyzed by ANOVA (α = 0.05), letters that differ are significantly different.

The tested adsorbers presented significant recovery differences, where the Diaion HP20 test achieved the highest (+)-zizaene titer from all the tested resins, followed by Amberlite XAD4, Amberlite XAD16N, Lewatit 1064MD and Amberlite IRA400 Cl tests (Figure 4A). Moreover, the (+)-zizaene recovery ratio from Diaion HP20 was similar to that obtained by the LLPPC control (92.5% vs. 94.4%, respectively) (Table S2).

Besides the Amberlite IRA400 Cl test, the rest of the adsorber tests recovered most of the (+)-zizaene amounts from the adsorbers (75.2%–92.5%), followed by the cell-free media (4.5%–18.7%) and in lower amounts from the cells (3.1%–6.1%; Table S2). As expected, only low (+)-zizaene amounts were measured from cell-free media by the negative control, confirming a loss of (+)-zizaene by volatilization. Besides, the highest amount of insoluble (+)-zizaene protein (inclusion bodies) was observed on the negative control, whereas most of the tested adsorbers and LLPPC showed low amounts of inclusion bodies (Figure S3). 

All of the tested adsorbers obtained similar cell growth between OD_600_ 3.4–3.9 and biomass 0.96–1.17 g_DCW_ L^−1^ from the *E. coli* TZS+MevZS strain (Figure 4B). On the other hand, the negative control (without extractant) had a lower cell growth when compared to all the other tests, with an OD_600_ of 2.7 and biomass of 0.7 g_DCW_ L^−1^, suggesting a toxic effect from the (+)-zizaene amounts in the culture broth. Because of the high recovery performance from the Diaion HP20 between adsorbers and comparable results with the LLPPC control, the Diaion HP20 was selected for further cultivations. 

### 2.3. Assessment of Organic Solvents for the Desorption of (+)-Zizaene 

After the screening of adsorbers for the SLPPC, an evaluation was done to analyze the elution performance of different organic solvents, which ideally should have a high partition coefficient and high selectivity towards (+)-zizaene [19]. Consequently, distinct solvents with high log P values were tested comprising decane, dodecane, pentane, ethyl acetate, isopropanol, isooctane, and acetonitrile (Table S1). 

**Figure 5:**
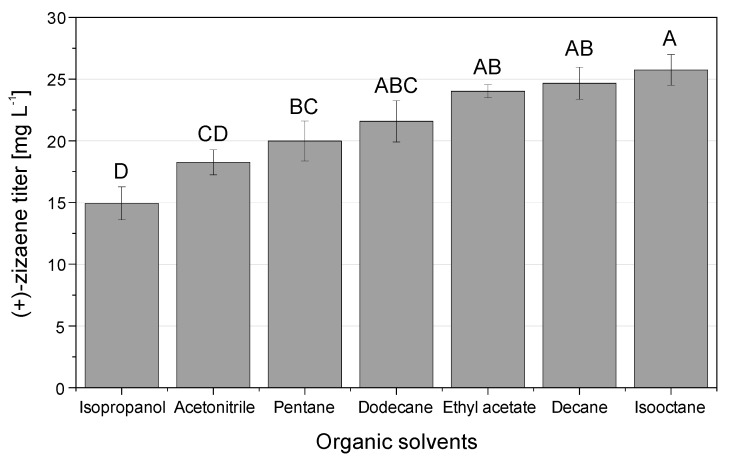
Elution performance of distinct organic solvents for the desorption of (+)-zizaene from the adsorber Diaion HP20. Data correspond to the mean of three independent experiments with error bars as SD. Data analyzed by ANOVA (α = 0.05), letters that differ are significantly different.

As evidenced in Figure 5, the organic solvent tests displayed significant desorption differences, with (+)-zizaene titers between 15–25 mg L^−1^ approximately. The eluents isooctane, decane, and ethyl acetate recovered the highest (+)-zizaene titers (25.7, 24.7, and 24.0 mg L^−1^, respectively), followed by dodecane, pentane, acetonitrile, and isopropanol. As a consequence, isooctane was selected as the elution solvent and further used for scale-up tests. 

### 2.4. Integration of In Situ Recovery of (+)-Zizaene to Fermentation at Bioreactor Scale

**Figure 6:**
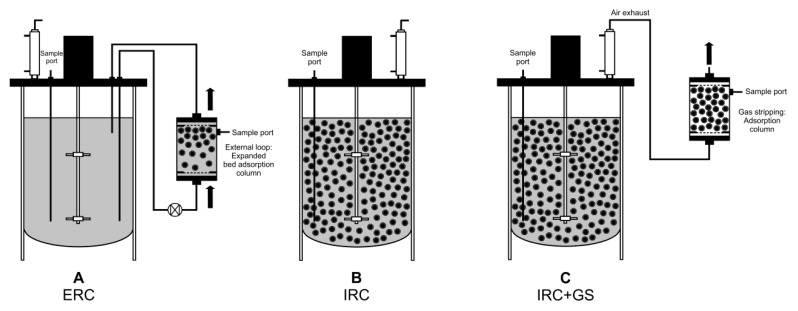
Diagram with the distinct bioreactor configurations used for the in situ recovery of (+)-zizaene. (**A**) External recovery: Adsorbers in expanded bed adsorption (EBA) column by an external loop. (**B**) Internal removal: Adsorbers inside the bioreactor vessel. (**C**) Internal removal with gas stripping: Adsorbers inside the bioreactor vessel and adsorption column coupled to the off-gas. Sample ports for adsorbers are indicated.

The fermentation was scaled up to 2 L stirred-tank bioreactors using the fed-batch cultivation method by feeding glucose continuously to reach HCDC. Because the use of liquid extractants is challenging for industrial-scale bioreactors [15,33], we tested the (+)-zizaene recovery with solid extractants. Thus, the external, internal, and internal with gas stripping in situ recovery configurations (Figure 6) were integrated to the fermentation with the best performing adsorber (Diaion HP20) and eluent (isooctane), in order to maximize product recovery and avoid cell toxicity, which could be more problematic at bioreactor scale due to the higher production of (+)-zizaene.

The Diaion HP20 adsorber was used at 50 g L^−1^, whereas higher amounts resulted in mixing problems and reading disturbances from the probes of the bioreactors. The optimal fermentation conditions for (+)-zizaene production at shake flask scale were previously determined [9] and used as the initial point for the bioreactor cultivations (ADM, pH 7.0), including induction at 20 °C to avoid formation of inclusion bodies from the ZS protein and to reduce volatilization of (+)-zizaene. Early induction was applied in all cultivations (OD_600_ 5–7) because late induction (OD_600_ > 15) was proven deleterious for the (+)-zizaene production, where most of the ZS protein was overexpressed as inclusion bodies (data not shown). 

### 2.5. Bioreactor Cultivation with an Integrated ERC 

The ERC was analyzed because external loops can facilitate product removal from large bioreactors and enable semi-continuous product recovery [15,38]. Moreover, the EBA chromatography was preferred as the extractant unit over the conventional packed-bed adsorption chromatography, because it allows operations at higher flow rates [40]. For this purpose, a stainless steel EBA column was built with the feature of a sampling port to ease the process monitoring and adsorber renovation (Figure 6A). The EBA was loaded with 75 g of Diaion HP20 and exchanged with 75 g of fresh resin every 24 h of operation.

**Figure 7:**
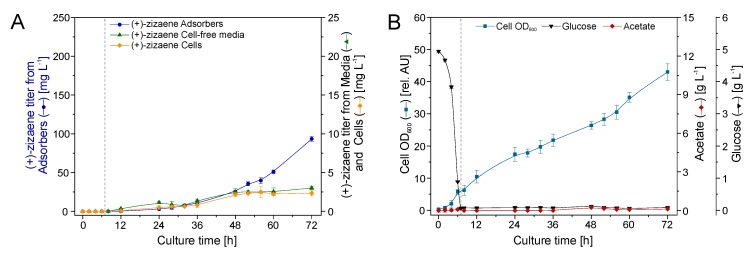
Production of (+)-zizaene (**A**) and growth kinetics (**B**) in 2 L bioreactor with external recovery configuration (ERC). Dotted line indicates division between batch and fed-batch stages.

The fed-batch stage started at 7 h of growth by lowering the temperature to 20 °C, and after 1 h the culture was induced with 0.5 mM ITPG (Figure 7B). Afterwards, the recirculation through the external loop (EBA) was initiated at a flow rate of 150 ml min^−1^. Thereafter, the production of (+)-zizaene and cell growth increased proportionally from 12 to 72 h of culture (Figure 7A), showing a coupling between both variables with a Pearson coefficient (PI) of 0.99. After 72 h of growth, the maximum production peak was observed, reaching a (+)-zizaene titer of 93.4 mg L^−1^ and an OD_600_ of 43.0. Therefore, the bioprocess improved the (+)-zizaene titer 2.5-fold and the cell growth 7.5-fold when compared to that of the LLPPC at shake flask scale. At the end of the cultivation (72 h), the recovery ratio (94.6%) from the adsorbers was similar to that obtained from the LLPPC test from Section 2.2 (94.4%), followed from the cell-free media (3.0%) and from the cells (2.4%). 

### 2.6. Bioreactor Cultivation with an Integrated IRC 

The IRC was evaluated as an alternative to improve the (+)-zizaene recovery further, whereas the configuration was designed to recover (+)-zizaene directly from the culture broth and improve the adsorption velocity (Figure 6B). To maintain the same amount of resin as the ERC, the bioreactor was loaded with 75 g of adsorbers. 

**Figure 8:**
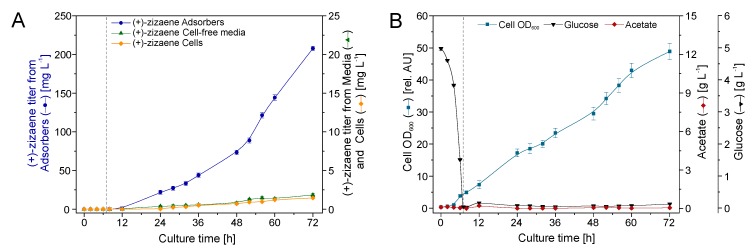
Production of (+)-zizaene (**A**) and growth kinetics (**B**) in 2 L bioreactor with internal recovery configuration (IRC). Dotted line indicates division between batch and fed-batch stages.

Similar to the ERC, a positive correlation between (+)-zizaene levels and cell growth was observed from 12 to 72 h (PI = 0.98) (Figure 8B). Moreover, the production of (+)-zizaene increased after 24 h, achieving the highest (+)-zizaene titer of 207.8 mg L^−1^ and cell growth (OD_600_ 48.9 and biomass 10.3 g_DCW_ L^−1^) at the end of the fermentation (Figure 8A). Thus, the IRC improved the (+)-zizaene titers 2.2-fold when compared to that of the ERC. In addition, the (+)-zizaene recovery ratio from adsorbers was improved to 98.4% when compared to the ERC and reduced the amounts of (+)-zizaene from cell-free media (0.9%) and cells (0.7%). 

### 2.7. Bioreactor Cultivation with an Integrated IRC+GS 

As discussed in Section 2.1, there was a loss of (+)-zizaene by volatilization at shake flask scale, which could be even higher at bioreactor scale due to the gassing of air through the bioreactor vessel. Therefore, the (+)-zizaene recovery from the off-gas of the bioreactor could be considered as an additional recovery source to enhance the accumulative (+)-zizaene recovery from the fermentation. For that, a variant from the IRC was performed with the addition of a column loaded with 75 g L^−1^ of adsorbers and installed in the off-gas of the bioreactor (Figure 6C).

**Figure 9:**
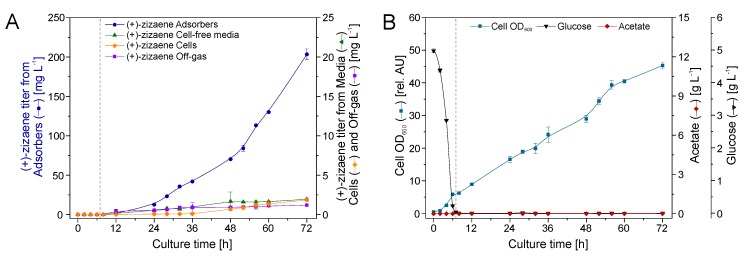
Production of (+)-zizaene (**A**) and growth kinetics (**B**) in 2 L bioreactor with internal recovery configuration with gas stripping (IRC+GS). Dotted line indicates division between batch and fed-batch stages.

Similar to the other bioreactor configurations, the correlation between cell growth and (+)-zizaene production on the IRC+GS was positive with PI = 95%, indicating a coupling between both variables. The results were similar to the IRC test, where after 72 h of culture, the maximum cell growth (OD_600_ 45.3 and biomass 9.7 g_DCW_ L^−1^) and (+)-zizaene production (203.4 mg L^−1^) were reached (Figure 9A,B). The (+)-zizaene amounts were not detected on the ethanol trap, demonstrating efficient adsorption of (+)-zizaene from the resins in the off-gas column with a (+)-zizaene recovery ratio of 0.6%. These low amounts of (+)-zizaene detected on the off-gas suggest an efficient (+)-zizaene trapping from the adsorbers in the culture broth, showing a recovery ratio from adsorbers of 97.6% and a low recovery ratio of 0.9% from both cell-free media and cells, similar to the IRC.

### 2.8. Accumulative (+)-Zizaene Production from the Bioreactor Configurations

**Figure 10:**
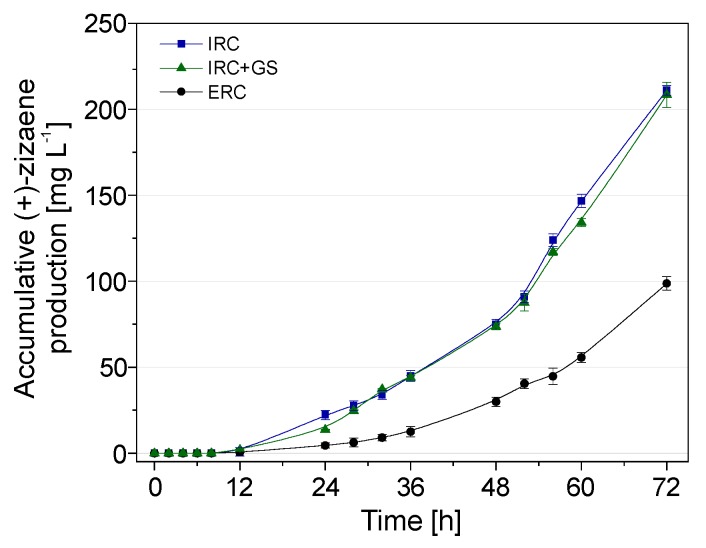
Accumulative production of (+)-zizaene at 2 L bioreactors by distinct in situ recovery configurations: External extraction (ERC), internal extraction (IRC), and internal extraction with gas stripping (IRC+GS). Data are the means of three sample replicates with error bars as SD.

The contribution to the accumulative (+)-zizaene amount from the IRC+GS was low (less than 1%). In consequence, the IRC and the IRC+GS showed similar accumulative (+)-zizaene levels during the course of the fermentation, whereas the difference between the ERC and both IRCs increased after 12 h of cultivation (Figure 10).

The comparison of performance between bioreactor configurations is summarized in Table 1. The accumulative (+)-zizaene titer and productivity between the IRC and IRC+GS were more than 2-fold higher than the ERC at 72 h of cultivation. Although differences between cell growth and product yield (Y_X/S_) were not so drastic between IRCs and ERC, the production of soluble ZS protein from the IRCs was roughly 4-fold higher when compared to the ERC, suggesting a relation between the soluble ZS protein and the (+)-zizaene levels. In consequence, the best configuration for the in situ recovery of (+)-zizaene at bioreactor scale was the IRC; achieving an accumulative (+)-zizaene titer of 211.13 mg L^−1^ and productivity of 3.2 mg L^−1^ h^−1^. Moreover, these results improved the (+)-zizaene titers 8.4-fold and productivity 3-fold when compared to those from our last report [10].

## 3. Discussion

For the microbial production of (+)-zizaene, significant product volatilization was observed when the recombinant *E. coli* strain was cultured without extractants, similar to that observed with the sesquiterpenes α-humulene [41]. Regardless of the low vapor pressure (5.1 kPa at 20 °C) and boiling point (288 °C) of (+)-zizaene, the volatilization of (+)-zizaene could be explained due to its low aqueous solubility (0.1289 mg L^−1^ [42]), which practically remains immiscible in the aqueous broth and tends to volatilize. As a solution, we tested the LLPPC at shake flask scale and improved the (+)-zizaene recovery and cell growth, reducing then the cell toxicity. Similar results were shown for the recovery of amorpha-4,11-diene, where the titers from the LLPPC tests were 8.5-fold higher than the controls without extractants [43]. Such improvements can be explained because the organic solvents extract the isoprenoids during culture due to hydrophobic interactions, and partition from the aqueous phase (culture broth) as demonstrated in many terpene studies [1,44,45]. 

Accordingly, the Diaion HP20 obtained the highest (+)-zizaene recovery ratio from all tested adsorbers. In comparison with the other adsorbers, Diaion HP20 has the largest pore size (290 Å) and pore volume (1.3 mL g^−1^) and it is used commonly for the adsorption of relatively large molecules such as small proteins. Although the sesquiterpene (+)-zizaene is not considered a large molecule (204.35 g mol^−1^), the large pores from the Diaion HP20 could possibly favor the (+)-zizaene adsorption. This idea could be supported due to the low recovery from the Lewatit 1064 MD, which has the lowest pore size of all the tested adsorbers (50 Å). Similar to our findings, the Diaion HP20 resin obtained the highest recovery ratio for the fragrant benzaldehyde (106.12 g mol^−1^) and L-phenylalanine (165.19 g mol^−1^) in bioreactor cultivations of *Pycnoporus cinnabarinus* [46], as well as for the prodigiosin-like red pigment (323 Da) from *Serratia sp*. KH-95 [47], whereas the Amberlite XAD16 adsorber (pore size 200 Å) showed the lowest recovery for both cases. 

Additionally, all of the tested adsorbers demonstrated higher cell growth when compared to the negative control without extractants. Such results were expected since it is known that most of the adsorbers do not affect cell growth due to their synthetic polymeric composition [37,48]. In addition, the low cell growth from the negative control suggests a toxic effect due to (+)-zizaene accumulation, similar to the toxic effect of linalool and linalool oxides accumulation on the fermentation of *Corynespora cassiicola* [27]. Eventually, further tests are required to measure the toxicity threshold of (+)-zizaene in fermentations. 

Concerning the screening of solvents for the desorption of (+)-zizaene, the results were as expected, where solvents with log P values higher than 0.73 eluted the highest sesquiterpene amounts due to their capability to trap hydrophobic compounds [15,19]. Although many studies used ethyl acetate [11,25] for terpene desorption, in our case we chose isooctane (log P = 3.80) because of the similar product recovery when compared to ethyl acetate but at a lower cost. In addition, isooctane has been proven suitable for the recovery of other terpenes such as limonene-1,2-diol produced by *Rhodococcus erythropolis* DCL14 [49]. Moreover, isooctane was used successfully for the extraction of (+)-zizaene on in vitro biotransformation reactions, as shown in our previous report [10]. 

To recover efficiently the (+)-zizaene at bioreactor scale, distinct ISPR configurations were integrated into the fermentation, using Diaion HP20 as adsorber and isooctane as elution solvent. Although these were chosen due to their recovery performance, other factors such as their low cost and ease for implementation were considered for the selection criteria. 

Initially, the ERC improved the (+)-zizaene production 2.5-fold when compared to that of the LLPPC at shake flask tests. Eventually, the minimal growth by feeding glucose maintained the acetate levels at a minimum, allowing the fermentation to reach a higher cell growth (OD_600_ of 43.0), and avoiding an overflow metabolism. Similar cell growth was also achieved by fed-batch fermentation of a metabolically-engineered *E. coli* strain for the production of (-)-α-bisabolol, fed with glycerol after 72 h of culture [14]. Besides, the external loop of the ERC allowed the semi-continuous product recovery, reaching (+)-zizaene titers of 93.4 mg L^−1^. Such improvements have also been obtained by the use of ERC in other terpenes studies, such as monoterpene carvone (225 mg L^−1^) [37] and diterpene cembratriene-ol (78.9 mg L^−1^) [50]. 

The (+)-zizaene production was enhanced 2.2-fold further by the use of the IRC when compared to the ERC. Besides, the (+)-zizaene amounts from cell-free media from the IRC were lower than the ERC. This demonstrates a higher (+)-zizaene recovery ratio from the IRC (98.4%) than the ERC (94.6%), which resulted in higher cell growth and soluble ZS protein synthesis, suggesting cell toxicity from (+)-zizaene accumulation in the culture broth of the ERC. This could be explained due to the residence time of the adsorbers on the culture broth, whereas in the ERC, the adsorbers have less time in contact with the culture broth (recirculation rate 6 h^−1^) than in the IRC, in which it resides constantly. Similar results were obtained for the production of prodigiosin-like red pigment from *Serratia sp.* KH-95, where the ERC obtained lower amounts than the IRC due to the lower contact of the adsorbers in the culture broth [51]. Consequently, terpenes need to be recovered rapidly from the culture broth before becoming volatilized. This was confirmed by the IRC+GS, in which less than 1% of the (+)-zizaene amount was volatilized due to the efficient (+)-zizaene recovery from the adsorbers inside the vessel (97.6%). Hence, the adsorption velocity plays an important role in the ISPR of terpenes. 

The IRC showed lower (+)-zizaene amounts from the cells than the ERC; demonstrating no correlation between cell growth and (+)-zizaene amount from cells. Possibly, the constant contact of the *E. coli* cells with the Diaion HP20 adsorbers from the IRC could improve the secretion and trapping of (+)-zizaene by an adsorption mechanism (chemisorption) as described in Figure 1. Similar behavior was observed in the production of a prodigiosin-like red pigment, where the compound was bound to the cell wall from *Serratia sp.* KH-95 and it was adsorbed towards the Diaion HP20 adsorbers, dispersed in the culture broth [51]. 

The contribution of the (+)-zizaene recovery from the adsorbers on the off-gas was lower than 1%, which demonstrates an efficient (+)-zizaene recovery from the adsorbers. For further scale-up studies of sesquiterpenes, the gas stripping recovery could be unnecessary when the IRC is used. Thus, the accumulative (+)-zizaene recovery from the IRC achieved titers of 211.1 mg L^−1^ and productivities of 3.2 mg L^−1^ h^−1^, which are similar to other IRC terpene bioprocesses, such as fusicocca-2,10(14)-diene (43 mg L^−^1, 0.6 mg L^−1^ h^−1^) [11], α-humulene (60.2 mg L^−1^, 2.5 mg L^−1^ h^−1^) [41], and carvone (198 mg L^−^1, 2.9 mg L^−1^ h^−1^) [37]. Moreover, our study demonstrated higher titers and productivities when compared to other LLPPC terpene bioprocesses, such as patchoulol (50 mg L^−1^, 0.65 mg L^−1^ h^−1^) [52] valerenadiene (62 mg L^−1^, 1.3 mg L^−1^ h^−1^) [53], and farnesol (135.5 mg L^−1^, 2.8 mg L^−1^ h^−1^) [54]. Consequently, the use of IRC with solid extractants is a promising alternative towards the scale-up of the microbial production of terpenes.

## 4. Materials and Methods 

### 4.1. Materials and Chemicals

Chemicals used in this study were of analytical grade. Polymeric adsorbers were Amberlite® IRA400 Cl, XAD4, XAD16N (Supelco, Bellefonte, PA, USA), Lewatit® 1064 MD (Lanxess, ‎Cologne, Germany), and Diaion HP20 (Mitsubishi Chemicals, Tokyo, Japan).

### 4.2. Strain and Pre-Cultures

The metabolically-engineered strain used in all the experiments of this research was the multi-plasmid *E. coli* Tuner TZS+MevZS strain, as described in our previous report [9]. 

All pre-cultures were prepared in 5 mL LB broth with 30 mg L^−1^ kanamycin and 34 mg L^−1^ chloramphenicol from glycerol stocks, and cultivated at 37 °C in a rotatory incubator at 150 rpm. For shake flask experiments, pre-cultures were grown overnight and inoculated to main cultures consisting of 35 mL of a modified Aparicio defined medium (ADM, [9]) with 5 g L^−1^ glucose in sealed glass-baffled shake flasks, to initiate at an OD_600_ of 0.1, and grown with the same conditions as mentioned before. Induction was performed when cultures reached OD_600_ 0.6–0.8 by lowering the temperature to 20 °C and adding isopropyl-β-D-thiogalactoside (IPTG) to a final concentration of 0.5 mM.

### 4.3. Product Volatilization and LLPPC Experiments

Shake flask cultures without extractants and induced for 24 h were centrifuged for 20 min (10,000 × g at 4 °C) and the supernatant was filtered through a 0.2 µm filter. Cell-free broth was transferred to sterile shake flasks and incubated at 20 °C and 150 rpm. Samples were taken from the cell-free broth, further extracted and terpene products were measured via gas chromatography coupled with a flame ionization detector (GC-FID).

For the LLPPC evaluation, cultures were prepared as in Section 4.2 and 10% isooctane (v/v) was added promptly after the addition of IPTG. No extractant was added to the negative control. Cultures were grown for 72 h and every 12 h samples were taken for growth kinetics and terpene analytics. 

### 4.4. Screening of Polymeric Adsorbers

The testing of adsorbers was performed using an in vivo method as described by Halka [11]. For that, distinct polymeric adsorbers (Table 2) were conditioned by washing them with water, isopropanol, isooctane, and finally water. After autoclaving shake flasks with 50 g L^−1^ of the respective adsorbers, 35 mL of ADM was added and inoculated with pre-culture broth to an initial OD_600_ of 0.1. Growth conditions and induction procedures were according to Section 4.2 and samples were analyzed after 24 h of induction. Terpene products were extracted from adsorbers, cell-free broth, and cells according to Section 4.7.3. A negative control without extractants was included, in which only cell-free broth and cells were analyzed. Identification of terpene products was carried out via gas chromatography with mass spectrometry (GC-MS) and quantification of (+)-zizaene was carried out via GC-FID. To measure the effect of the tested adsorbers on cell growth, the OD_600_ and the dry cell weight (DCW) biomass were analyzed from the distinct resins. The (+)-zizaene recovery ratio from adsorbers was calculated as the (+)-zizaene titer from adsorbers between the accumulative (+)-zizaene titer (recovered from adsorbers, cells, and cell-free media). Data sets were analyzed by ANOVA according to Section 4.8.

### 4.5. Evaluation of Organic Solvents for the (+)-Zizaene Desorption

Organic solvents with a high log P value were evaluated for the desorption of (+)-zizaene comprising decane, dodecane, isooctane, ethyl acetate, isopropanol, acetonitrile, and pentane (Table S1). The Diaion HP20 adsorbers were prepared as described in Section 4.4 and the tested organic solvents were used respectively for the conditioning of adsorbers. 

Microbial cultures were prepared as described in Section 4.2 without extractants and after 48 h of cultivation, 2 mL of culture broth was transferred to sterile vials with 0.5 g of Diaion HP20 adsorbers. Afterwards, vials were incubated for 4 h at 20 °C and 150 rpm. Furthermore, the culture broth was discarded and adsorbers were extracted with the tested solvents as described in Section 4.7.3 (+)-Zizaene concentrations were measured via GC-FID and statistical analyses were carried out according to Section 4.8.

### 4.6. Bioreactor Cultivations with In Situ Recovery of (+)-Zizaene 

Pre-cultures were prepared as described in Section 4.2, followed by a third pre-culture that consisted of a 100 mL ADM shake flask culture. After 12 h of cultivation, pre-cultures were inoculated to bioreactors to an OD_600_ of 0.3–0.4.

2 L stirred-tank bioreactors (Biostat A+, Sartorius, Göttingen, Germany) were used for the cultivations, with 1.5 L working volume consisting of an ADM with 5 g L^−1^ glucose and respective antibiotics. The pH was controlled automatically with 1 M HCl and 25% NH_4_OH (*v*/*v*) solutions. The Diaion HP20 adsorbers were conditioned according to Section 4.4 with isooctane as the solvent.

#### 4.6.1. IRC Bioreactor

The IRC was prepared with 50 g L^−1^ of Diaion HP20 dispersed in the culture broth, inside the bioreactor vessel. The IRC with gas stripping (IRC+GS) was carried out similarly to the IRC and a 200 mL column was added to the off-gas line loaded with 75 g of Diaion HP20, followed by an ethanol trap cooled with dry ice and two 0.2 µm sterile filters. 

#### 4.6.2. ERC Bioreactor

The ERC was configured without extractants inside the bioreactor vessel, and the culture broth was recirculated to an external recovery loop through a stainless steel EBA column of 200 mL inner volume with 75 g of Diaion HP20 as the fluidized bed. For that, a silicone tubing of 4 mm inner diameter (i.d.) was connected to the EBA column (4.6 cm i.d., 12 cm length for inner chamber) with outlets of 4 mm i.d. and a sampling port on the lateral side. The EBA column had two stainless steel meshes (6 cm diameter of 500 µm) on both terminal sides, fixed with gaskets to recirculate cells and media through the resins while retaining the adsorbers inside the column. The external loop operated continuously at a flow rate of 150 mL min^−1^ (recirculation rate = 6 h^−1^) by a SciLog Expert peristaltic pump (Wisconsin, USA). 

For all bioreactor configurations, the batch cultivation settings were dissolved oxygen >30%, agitation 400–700 rpm, temperature 37 °C, pH 7.0, and gas flow rate 1.0 vvm. When the glucose was exhausted, the fed-batch stage was initiated by dropping the temperature to 20 °C and initiating the feeding. The fed-batch medium was composed of glucose 100 g L^−1^, NaCl 1.2 g L^−1^, CaCl_2_ 0.14 g L^−1^, MgSO_4_·7H_2_O 0.6 g L^−1^, FeSO_4_·7H_2_O 0.001 g L^−1^, and CuSO_4_·5H_2_O 0.001 g L^−1^ with respective antibiotics. The induction was performed after 1 h of feeding by adding 0.5 mM IPTG and in the case of the ERC, the recirculation towards the external loop was activated. Samples for growth kinetics, soluble ZS protein, and terpene analysis were properly taken, measured according to Section 4.7, and plotted with Origin 9.5.5. (Northampton, MA, USA).

### 4.7. Analytical procedures 

#### 4.7.1. Growth Kinetics Analysis

To assess the growth kinetics from cultivations, the cell growth was analyzed by measuring the optical density from fermentation samples at 600 nm using a Biochrom Libra S50 UV-Vis spectrophotometer (Cambridge, UK) and biomass by the dry cell weight method. Glucose consumption was measured from cell-free broth samples with the Biochemistry Analyzer YSI 2900 (Yellow Springs, Greene County, OH, USA). Acetate was measured via high-pressure liquid chromatography as described elsewhere [55]. 

#### 4.7.2. Soluble ZS Protein Fraction Analysis

Broth samples were normalized to an OD_600_ of 2.0 and extracted as described in our previous report [9]. Soluble ZS protein fractions were analyzed on a 10% sodium dodecyl sulfate polyacrylamide gel electrophoresis with a calibration curve of bovine serum albumin. Quantification of soluble ZS protein was performed by a densitometric method [56,57], measuring the intensity of the ZS protein bands at 78 kDa from stained gel images by the GelAnalyzer 2010 (developed by Istvan Lazar).

#### 4.7.3. Sample extraction

Samples for terpene analyses were extracted from distinct sources during cultivations. Samples from cell-free media, known also as supernatant, were prepared by transferring 2 mL of culture broth to 10 mL glass vials. After centrifugation for 5 min at 10,000× *g*, the supernatant was transferred to other glass vials and extracted vigorously thrice with 0.5 mL of isooctane. Organic phases were obtained by centrifugation and transferred to GC vials for (+)-zizaene measurements via GC-FID. 

Samples from cells were prepared similar to Section 4.7.2. After extracts were ultrasonicated, 300 µL of isooctane was added to 300 µL of cell extract and extracted vigorously. Organic phases were separated by centrifugation and transferred to GC vials for (+)-zizaene measurements via GC-FID. 

Samples from adsorbers were extracted vigorously three times by transferring 300 mg of resins to 10 mL glass vials and adding 1 volume of isooctane (or tested solvent for Section 4.5). Organic phases were transferred to GC vials for further product identification via GC-MS and (+)-zizaene quantification via GC-FID.

#### 4.7.4. GC-MS analysis

For the identification of terpene products from the adsorber screening test (Section 4.4), the extracted samples were analyzed by an Agilent 7890B GC-MS system (Santa Clara, CA, USA). Samples of 0.5 µL were injected into the GC-MS equipped with a VF-WAXms capillary column (0.25 mm i.d. × 0.25 µm thickness × 30 m length; Agilent, Santa Clara, CA, USA) by the on-column mode with helium 5.0 as the carrier gas at a constant gas flow of 1 mL min^−1^ and injector temperature of 230 °C. The oven program comprised 3 steps: (1) 40 °C, 3 min; (2) 40–230 °C, 10 °C min^−1^; (3) 10 min hold. The scan range was set to 33–300 *m/z* and the ionization energy to 70.0 eV. Product identification was carried out by comparing mass spectra of samples with authentic standards obtained from the VEO and references from the mass spectral NIST 14 database. 

#### 4.7.5. GC-FID analysis

Quantification of (+)-zizaene was done with a GC-2010 plus Shimadzu system coupled with a flame ionization detector (Kyoto, Japan). Samples of 1 µL were injected to the GC-FID with an injector temperature of 240 °C on splitless mode. Oven program was set with the following steps: (1) 40 °C, 20 s; (2) 40–200 °C, 10 °C min^−1^; (3) 0.5 min hold; (4) 200–230 °C, 30 °C min^−1^; (5) 2 min final hold. The quantification of (+)-zizaene was calculated as α-cedrene equivalents by a calibration curve of α-cedrene (standard grade) due to the lack of a commercial (+)-zizaene standard, as demonstrated in a previous report [10].

### 4.8. Statistical Analysis

Data from Section 4.4 and Section 4.5 were analyzed by analysis of variance (ANOVA) and mean comparison tests to assess statistical differences. Adsorbers and organic solvents were used as factors respectively and (+)-zizaene titer was used as the response variable. Data sets were analyzed by Minitab 16 (Pennsylvania, USA) with the ANOVA module and the Bonferroni test was applied for mean comparison test with a 95% confidence level. 

## 5. Conclusions

The results achieved in this study demonstrated the improvement of the microbial production of (+)-zizaene compared to previous studies by enhancing the recovery of (+)-zizaene. Initially, the loss of (+)-zizaene by volatilization was measured and further reduced by LLPPC at shake flask scale. Furthermore, the Diaion HP20 resin obtained the highest (+)-zizaene recovery after screening distinct adsorbers by SLPPC. After evaluating distinct solvents for the desorption process, the isooctane was selected as a suitable eluent and the SLPPC reached (+)-zizaene titers comparable to those obtained by the LLPPC. The scale-up to bioreactors by integrated product recovery configurations improved dramatically the (+)-zizaene production, whereas the IRC demonstrated higher (+)-zizaene titers (211.13 mg L^−1^) and productivities (3.2 mg L^−1^ h^−1^) than the ERC. Consequently, the successful application of ISPR proved a greener extraction method, which reutilizes the extractant material (polymeric adsorbers), reduces the number of extraction reagents (only one solvent is required), reduces the energy input and quantity of chemical wastes, and improves the recovery ratio of (+)-zizaene over 98%.

## Figures and Tables

**Table 1:** Comparison of the bioprocess-performance variables between distinct bioreactor configurations for the in situ recovery of (+)-zizaene after 72 h of growth.

Variable	ERC	IRC	IRC+GS
Accumulative titer (mg L^−1^)	98.78 ± 3.87	211.13 ± 1.97	208.41 ± 7.29
Titer from adsorbers (mg L^−1^)	93.42 ± 3.30	207.84 ± 1.68	203.44 ± 6.89
Productivity (mg L^−1^ h^−1^)	1.50 ± 0.06	3.20 ± 0.03	3.16 ± 0.11
Y_P/X_^1^ (mg_zizaene_ g_DCW_^−1^)	12.32 ± 0.56	20.45 ± 6.98	21.41 ± 2.29
Y_X/S_^2^ (g_DCW_ g_glucose_^−1^)	0.22 ± 0.003	0.28 ± 0.11	0.30 ± 0.02
Y_P/S_^3^ (mg_zizaene_ g_glucose_^−1^)	2.69 ± 0.08	5.82 ± 0.09	6.52 ± 0.18
Soluble ZS protein (mg L^−1^)	35.42 ± 3.20	143.20 ± 2.95	129.80 ± 6.51
Adsorber recovery ratio (%)	94.60 ± 0.14	98.40 ± 0.21	97.60 ± 0.12

^1^ Y_P/X_: product/biomass yield. Y_X/S_: ^2^ Biomass/substrate yield. ^3^ Y_P/S_: product/substrate yield. Data correspond to the mean of three sample replicates with ± SD.

**Table 2:** Main properties of the polymeric adsorbers used for the in situ recovery of (+)-zizaene ^1^.

Properties	Amberlite IRA400 Cl	Lewatit 1064 MD	Amberlite XAD16N	Amberlite XAD4	Diaion HP20
Particle size (µm)	600–750	440–540	560–710	490–690	250–800
Mean pore size (radius) (Å)	100	50	200	100	290
Surface area (m^2^ g^−1^)	‒	800	800	750	590
Pore volume (mL g^−1^)	‒	1.2	0.55	0.5	1.3
Particle density (mg L^−1^)	1.06–1.09	1.02	1.02	1.02	1.01
Functional groups	Dimethyl ethanol ammonium	‒	‒	‒	‒
Ionic form	Basic anion exchange	Non-ionic	Non-ionic	Non-ionic	Non-ionic

^1^ Information obtained from supplier data sheets.

## Supplementary Materials

The following are available online: Figure S1: Terpene profile between distinct polymeric adsorbers; Figure S2: Mass spectra of terpene products; Figure S3: Comparison of soluble and insoluble ZS protein amounts between distinct polymeric adsorbers; Figure S4: Off-line analytics from bioreactor configurations; Table S1: Physicochemical properties of the organic solvents used for the desorption of (+)-zizaene; Table S2: Recovery ratio of (+)-zizaene from distinct polymeric adsorbers.
