# Supplementary material for: Improved Production and In Situ Recovery of Sesquiterpene (+)-Zizaene from Metabolically-Engineered E. coli
Source: Molecules. 2019 Sep 15;24(18):3356. doi: 10.3390/molecules24183356 (PMC6767195; doi:10.3390/molecules24183356)
Supplement: Supplementary file 1 [file molecules-24-03356-s001.pdf]

Supplementary Materials

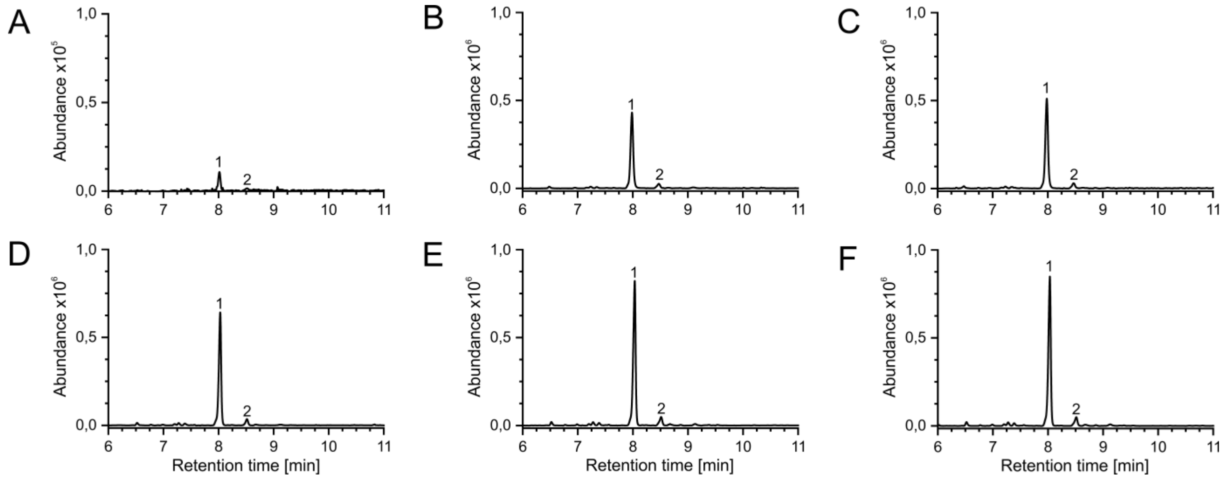

Figure S1. Comparison of the terpene profile between distinct polymeric adsorbers by GC-MS used for the ISPR on the cultivation of *E. coli* TZS+MevZS strain after 24 h of growth. (A) Amberlite IRA-400 chloride. (B) Lewatit 1064 MD. (C) Amberlite XAD16N (D) Amberlite XAD4 (E) Diaion HP20. (F) LLPPC. Peak identification: (1) (+)-zizaene, (2) β-acoradiene.

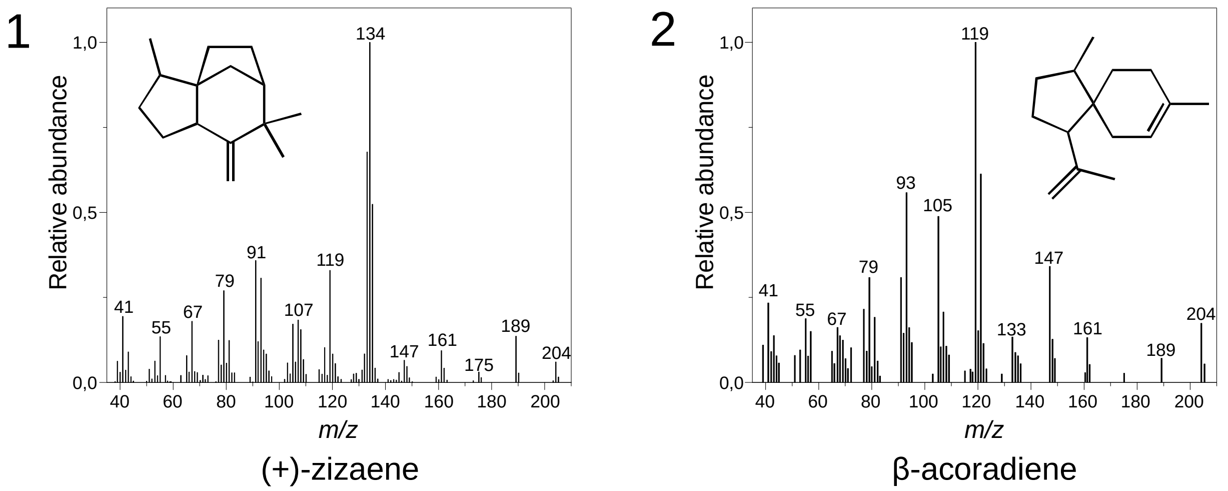

Figure S2. Mass spectra for the identification of the terpene products extracted by polymeric adsorbers from the cultivation of the *E. coli* TZS+MevZS strain. (1) (+)-zizaene (RT:1618). (2) β-acoradiene (RT: 1669).

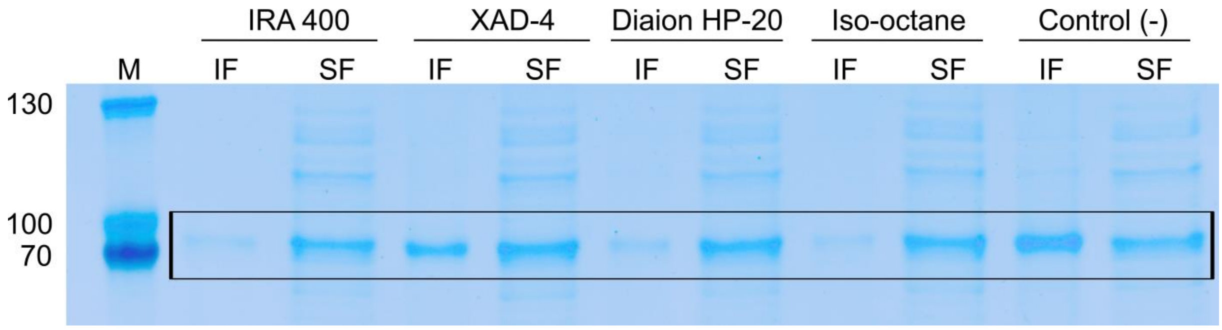

Figure S3. Analysis by 10% SDS-PAGE of soluble (SF) and insoluble (IF) ZS protein fractions, cultured with different hydrophobic adsorbers after 48 h of growth. (M) Molecular marker.

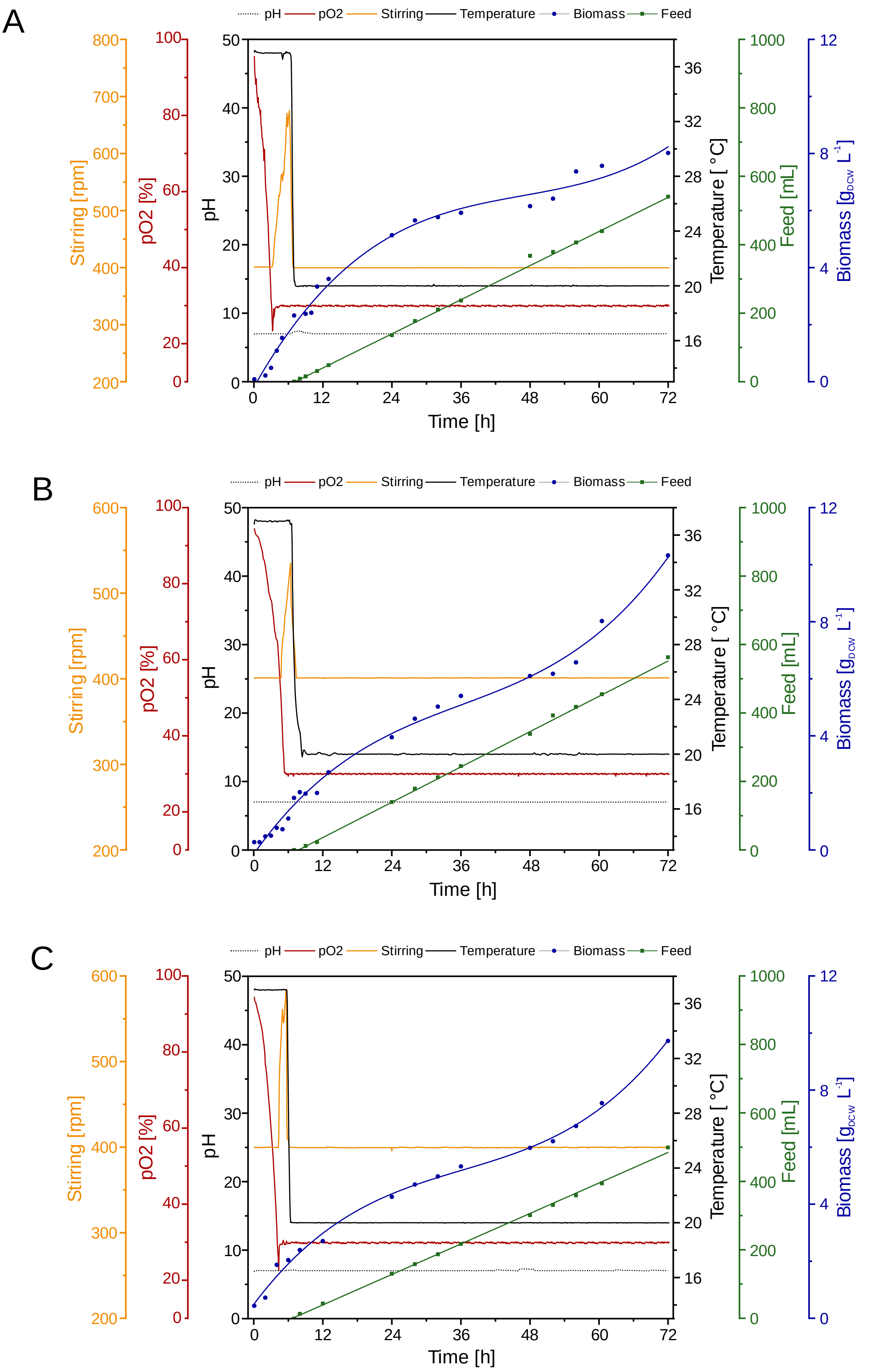

Figure S4. Off-line analytics (stirring, dissolved oxygen (pO2), pH and termperature), cell growth (biomass) and feeding from fermentations in 2 L bioreactors with in situ recovery configurations for (+)-zizaene. (A) ERC (B) IRC (C) IRC+GS.

Table S1. Physicochemical properties of the organic solvents used for the desorption of (+)-zizaene[1]

Solvent	MW [g mol⁻¹]	Boiling point [°C]	Vapor pressure [kPa]	Aqueous solubility (mg mL⁻¹)	Log ($P_{octanol}$)
Isopropanol	60.05	82.3	4.4 at 20 °C	100 at 22 °C	0.05
Acetonitrile	41.02	81.60	9.9 at 25 °C	100 at 22.5 °C	-0.34
Pentane	72.15	36.00	53.3 at 18.5 °C	1 at 21 °C	3.39
Dodecane	170.2	216.3	0.018 at 25 °C	1 at 25 °C	6.1
Ethyl acetate	88.05	77.10	10 at 20 °C	50 to 100 at 21 °C	0.73
Decane	142.17	174.10	0.17 at 25 °C	1 at 21 °C	5.01
Isooctane	114.14	99.20	5.1 at 20 °C	$1.96x10^{-05}$ M (None) at 21 °C	3.80

[1] Data obtained from PubChem [1]

Table S2. (+)-Zizaene recovery ratio from distinct polymeric adsorbers after 48 h of growth from the *E. coli* TZS+MevZS strain

Adsorbers	Cells	Media	Adsorbers
Control (-)[a]	36.2±1.9%	63.8±1.9%	-
Amberlite IRA400 Cl	53.9±6.9%	41.9±6.1%	4.2±0.8%
Lewatit 1064 MD	6.1±2.9%	18.7±7.6%	75.2±10.5%
Amberlite XAD16N	3.1±1.2%	13.1±5.2%	83.8±6.4%
Amberlite XAD4	4.5±2.3%	6.3±0.4%	89.2±2.7%
Diaion HP20	3.1±2.4%	4.5±1.2%	92.5±1.4%
LLPPC	2.0±1.8%	3.6±0.7%	94.4±2.0%

[a]Negative control cultured without extractant.
Data are the mean of the (+)-zizaene recovery ratio of three replicates with ± SD.
